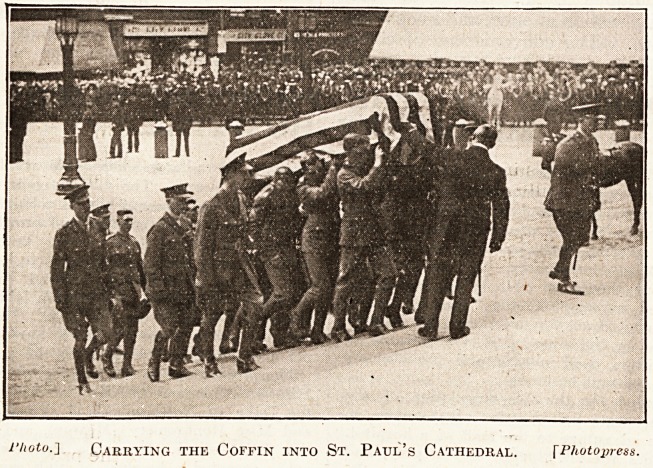# The Late General Gorgas, K.C.M.G., D.S.M.

**Published:** 1920-07-17

**Authors:** 


### THE LATE GENERAL GORGAS, K.C.M.G., D.S.M.

### An Impressive Service at St. Paul's.

ON Friday, July 9, at noon, was held the funeral service of Major-General William Crawford Gorgas, K.C.M.G., D.S.M., of the United States Army. Following a really imposing military procession, in which the body was brought from the Queen Alexandra Hospital, Millbank, the ceremony took place in St. Paul's Cathedral at noon. A salute of thirteen guns, fired at minute intervals in Hyde Park, was added to the many tokens of sincerity with which London and its vast crowds paid a last tribute to a man whose work for the well-being of mankind is realised the world over.

The coffin, covered with the Stars and Stripes and bearing a single and beautiful wreath of white flowers, was carried on one of the gun-carriages of the Royal Horse Artillery, and was preceded by the magnificent band of the Coldstream Guards. Six military pall-bearers were present, namely, Major-General Sir W. G. Macpherson, Major-General H. P. Blenkinsop, Major-General Sir G. B. Stanistreet, Colonel O. L. Robinson, Colonel Inge, and Colonel J. R. McMunn, and an escort drawn from the staff of the 2nd Guards' Brigade, three squadrons of the 2nd Life Guards, the 3rd Battalion Coldstream Guards, and the 1st Battalion Irish Guards, were under the command of Brigadier-General T. McC. Steele. The procession, in which, too, General Gorgas's charger followed the gun-carriage, proceeded along the Victoria Embankment, and thence reached St. Paul's shortly before twelve o'clock.

*Photo.*] CARRYING THE COFFIN INTO ST. PAUL'S CATHEDRAL. [*Photopress.*

IN ST. PAUL'S CHURCHYARD.

The sight in the Churchyard was most impressive. Great crowds had collected from all quarters and paid their silent respects to the memory of the American's great work. Amid a stillness, broken only by the sad strains of the Dead March from *Saul*, the mounted and unmounted troops, wheeling to right and left, completely ringed the Churchyard, and as the last arrived and rested on reversed arms the great bell of St. Paul's chimed the hour of noon. The coffin was borne up the steps of the famous Cathedral, met by the Dean and clergy, and thereupon a second procession gathered, now including as pall-bearers the United States military and naval attachés, Sir Anthony Bowlby (President of the Royal College of Surgeons), Surgeon-General Sir R. H. Charles, Brigadier-General John Finney, of the United States Medical Service, Lieut.-Col. Sir J. K. Fowler (Colonial Office), Surgeon Rear-Admiral Sir R. Hill (Director-General of the Medical Department of the Navy), Sir G. H. Makins, Dr. Charles Mayo, of Rochester, U.S.A., Sir Norman Moore, Sir H. D. Rolleston, Professor W. J. R. Simpson, and Mr. H. S. Wellcome.

The procession, then entering the Cathedral, came before a distinguished congregation, assembled beforehand. The King was represented by Lieut.-General Sir T. H. J. C. Goodwin, Queen Alexandra by Colonel Sir Henry Streatfield, the Duke of Connaught by Colonel Sir Edward Worthington, and among other well-known people could be noticed the American Ambassador, Dr. Addison, and many high officials of the British Government offices, of the International Red Cross Societies, and of many others. The South American Republics were practically all represented, and it is impossible to imagine a more distinguished and varied gathering of men of military, naval, and civilian fame from every quarter of the globe.

THE CATHEDRAL SERVICE.

The Guardsmen bearers laid the coffin in the catafalque under the dome, surrounded by six tall candlesticks and magnificent floral decorations. The singing of the choir which had led the procession up the nave now changed into Psalm xc., "Lord, Thou hast been our refuge." The first hymn was "Nearer, my God, to Thee," and the lesson, from I. Corinthians xv., 20-58, was read by the Dean, who was assisted in the service by the Sub-Dean, the Rev. W. P. Besley. Then the second hymn, "Lead, kindly Light," was sung, and the service ended with "The Battle Hymn of the Republic," the beautiful music of which, as one report of the ceremony comments, had for long disappeared from the memory of London until the war brought it back with a deepened and more intimate meaning. As the body was carried from the Cathedral the impressive strains of Chopin's *Marche Funèbre* brought to a close a most memorable service, at which those present must one and all have felt deeply the great loss which they and the world had sustained, and an appreciation of the magnificent achievements of a great man's life. America may be justly proud of the work he has accomplished.

## Figures and Tables

**Figure f1:**